# Retrograde NaviAid Enteroscopy-Assisted Resection of Distal Small Bowel Hamartomatous Polyps

**DOI:** 10.7759/cureus.11962

**Published:** 2020-12-07

**Authors:** Ayusa Sinha, Amitpal S Johal, Ansh Khurana, Puneet Basi, Harshit S Khara

**Affiliations:** 1 Division of Gastroenterology and Nutrition, Geisinger Health System, Danville, USA; 2 Department of Gastroenterology, UPMC Susquehanna Health, Williamsport, USA

**Keywords:** naviaid, hamartomatous polyp, peutz jeghers, retrograde enteroscopy, device-assisted enteroscopy, push enteroscopy, small bowel polyp, small bowel obstruction, symptomatic anemia, malignancy

## Abstract

Hamartomatous polyps are gastrointestinal tumors that may present with small bowel obstruction requiring surgical resection, while others may present earlier as symptomatic anemia prior to becoming an obstructing mass. Video capsule endoscopy has enhanced the early detection of small bowel lesions. However, endoscopic interventions especially for distal small bowel lesions are limited due to long procedure times, technical challenges in achieving depth of insertion, and the requirement of specialized deep enteroscopy equipment with advanced endoscopy training, which are not always available. Therefore, surgical intervention is often required. NaviAid-assisted enteroscopy, a novel thorough-the-scope balloon, results in deep anterograde and retrograde intubation of the small intestine using standard colonoscope and can be used for rapid therapeutic intervention. We present two cases of distal small bowel hamartomas which were resected via retrograde NaviAid-assisted enteroscopy, thus, preventing surgery.

## Introduction

Hamartomatous polyps are rare gastrointestinal polyps, which are often associated with Peutz-Jeghers syndrome and Cowden’s disease. The prevalence of these polyps is unknown because they are mostly asymptomatic. Patients who have these polyps may present with small bowel obstruction requiring resection [1,2]. With the advent of capsule endoscopy, diagnostic evaluation of the small bowel has improved significantly. Yet, endoscopic therapeutic interventions remain limited to anterograde push enteroscopy or anterograde device-assisted enteroscopy with balloon or spiral-overtube. These procedures are time-consuming and require specialized training and equipment, making surgical interventions necessary in many cases. NaviAid is a novel balloon-assisted enteroscopy device using a through-the-scope balloon with a standard colonoscope, without the need of cumbersome overtubes. This allows for deep intubation of the small intestine with reduced procedure times. Recent studies have demonstrated the safety and feasibility of this device in routine deep enteroscopy practice [3,4]. We present two unique cases in which NaviAid-assisted retrograde enteroscopy was used for resection of distal small bowel hamartomas, which would have been difficult to reach with other deep enteroscopy devices, thus preventing the need for bowel resection.

## Case presentation

**Figure 1:**
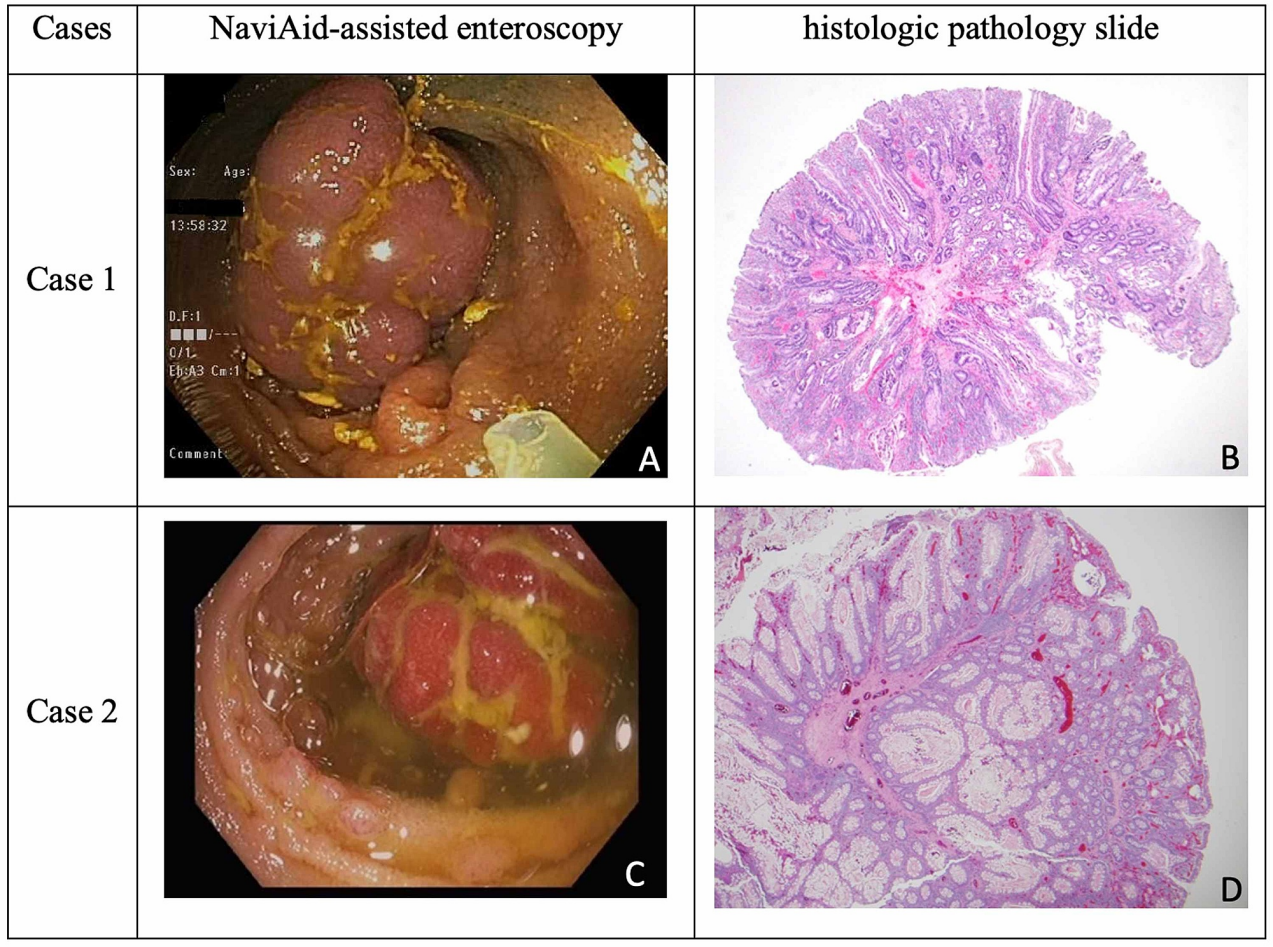
NaviAid-assisted enteroscopy and histologic path slide images of small bowel polyps resected from Case one and Case two

Case one is a 30-year-old female with no significant past medical history who presented to our hospital with diarrhea and melena, associated with dizziness, fatigue, and anemia, with hemoglobin of 8.0 (normal 12.0 - 15.3 g/dL). There was no family history of gastrointestinal malignancy. She had a 10-pack year history of cigarette smoking and consumed alcohol socially. Her vital signs were stable, and her physical exam was normal except for left upper quadrant tenderness on deep palpation. She underwent an upper endoscopy that revealed mild gastritis; biopsies were negative for *H. pylori*. Subsequent colonoscopy with terminal ileal intubation was normal. She then underwent a small bowel capsule endoscopy which showed an ulcerated 25 mm polypoid lesion in the distal ileum, approximately 10 cm upstream from the ileocecal valve. This finding prompted a follow up NaviAid-assisted retrograde enteroscopy, which revealed a partially obstructing mass in the distal ileum (Figure 1A). Endoscopic mucosal resection was performed, and the lesion was retrieved en-bloc successfully. Histology confirmed it to be a hamartomatous polyp of a kind associated with Peutz-Jeghers Syndrome (Figure 1B).

**Figure 2:**
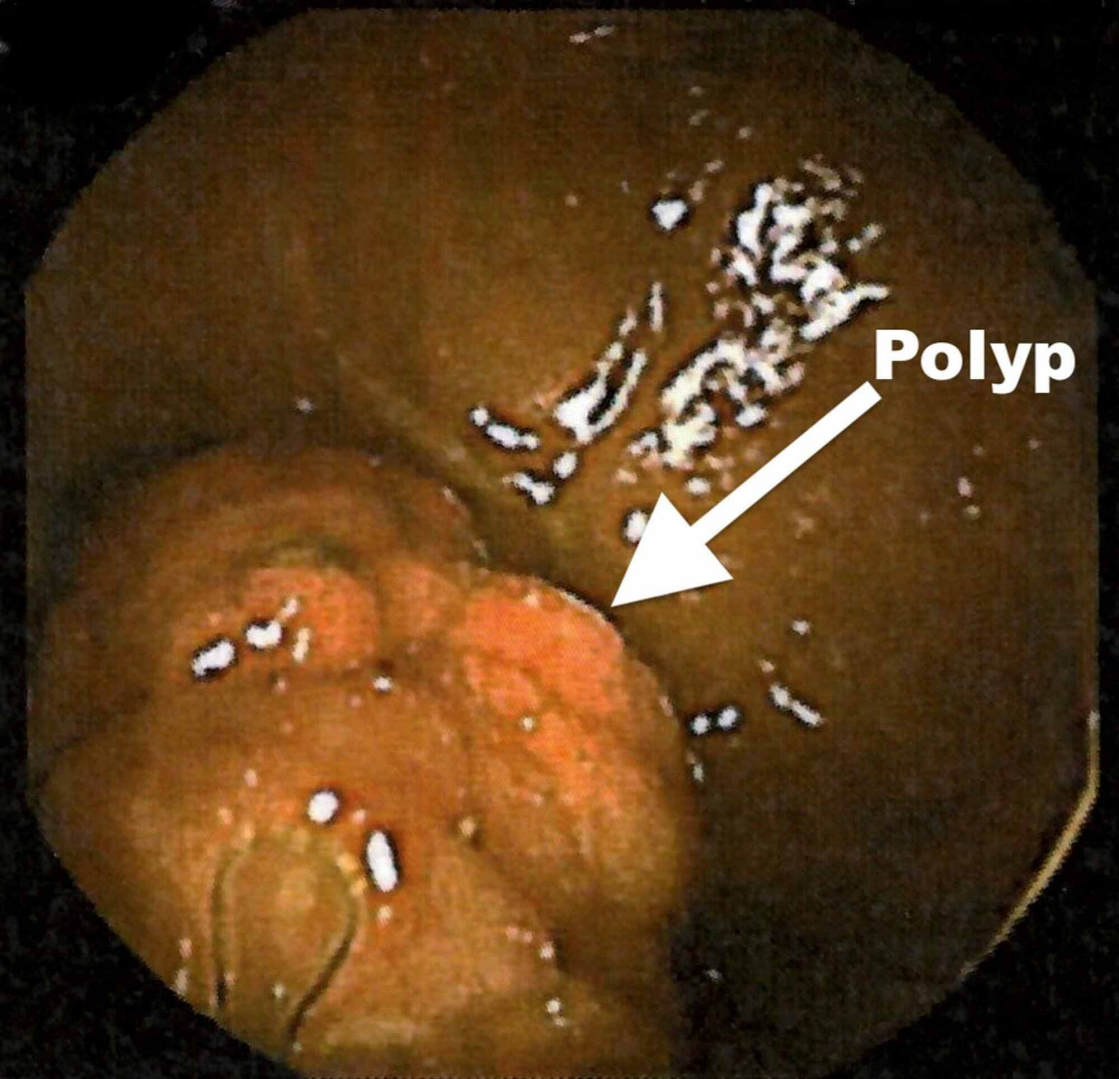
Capsule endoscopy image of distal ileal polyp in Case two

Case two is a 46-year-old male with a known history of Peutz-Jeghers syndrome, diagnosed at age four years when he presented with bowel obstruction requiring bowel resection. At age 16 years, he developed bowel obstruction again and underwent another resection. During surveillance colonoscopy and endoscopy two years prior to presentation multiple colonic and gastric polyps were identified and endoscopically removed. A surveillance small bowel capsule endoscopy showed a semi-pedunculated 20 mm polyp in the distal ileum (Figure 2). The patient was referred for small bowel enteroscopy and endoscopic resection. We performed a NaviAid-assisted retrograde enteroscopy. The distal ileal polyp was removed en-bloc (Figure 1C) successfully with endoscopic mucosal resection and confirmed to be a hamartomatous polyp (Figure 1D).

## Discussion

**Figure 3:**
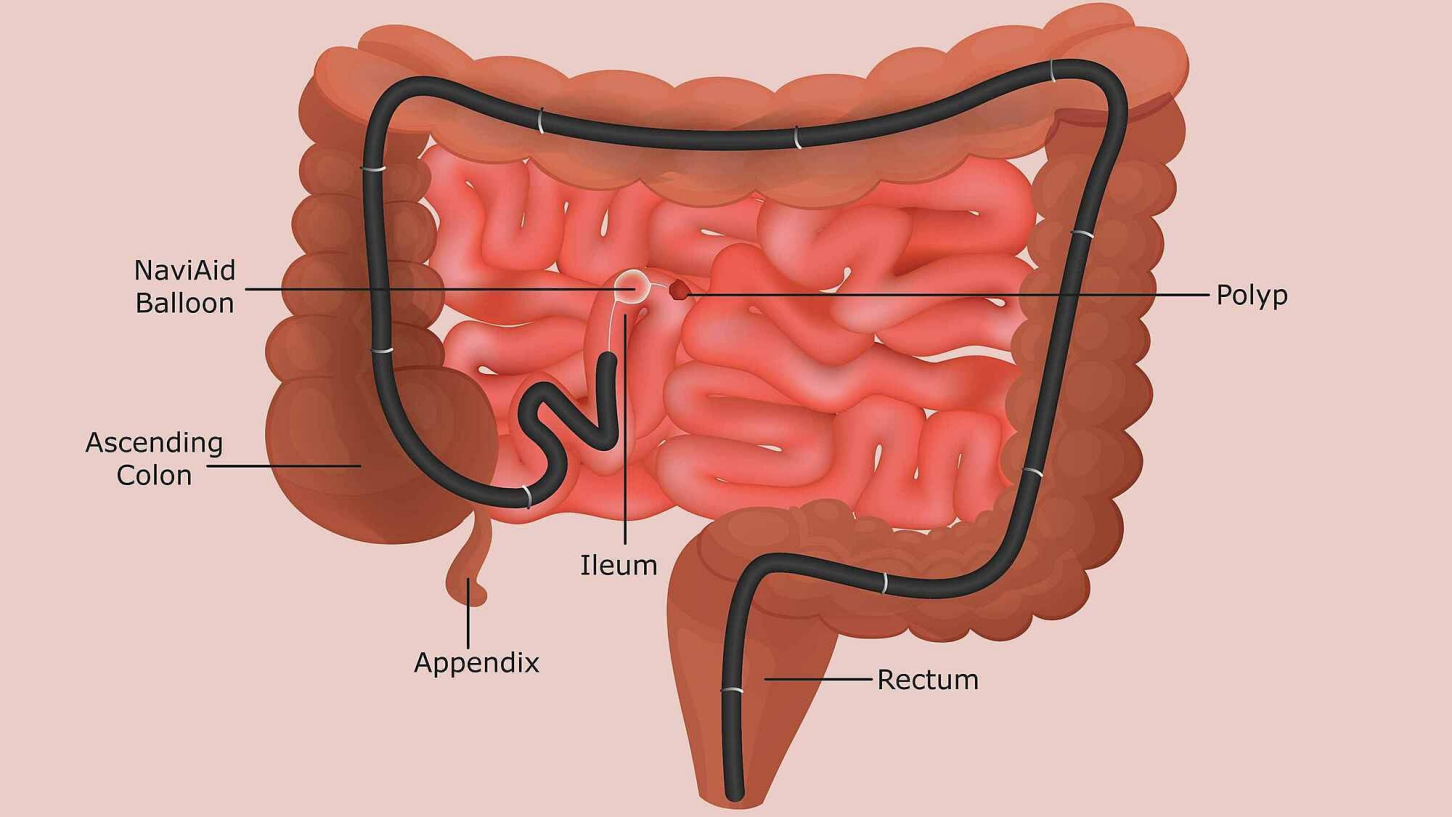
Diagram illustrating the Naviaid system as it traverses the colon and into the ileum

Hamartomatous polyps are often associated with polyposis syndromes such as Peutz-Jeghers and can present initially as a small bowel obstruction as seen in Case two. Hamartomatous polyps can also present insidiously with intermittent bleeding resulting in anemia as seen in Case one. Both patients underwent small bowel evaluation by capsule endoscopy which identified large polyps in the distal small bowel requiring further intervention. Previously therapeutic intervention for hamartomatous polyps was limited to anterograde push enteroscopy, device-assisted enteroscopy, or surgery for small bowel resection as seen in Case two. NaviAid-assisted retrograde enteroscopy is a novel technique that involves using a through-the-scope balloon via standard colonoscopes without the need for specialized overtubes or dedicated scopes, and thus can be used “on-demand” during a routine procedure (Figure 3). Two recent studies indicated that retrograde enteroscopy with NaviAid had reduced procedure time of 23 minutes, compared to 43 minutes for device-assisted enteroscopy [3,5]. Both our patients tolerated the procedure well, without any complications such as perforation, bleeding, acute pancreatitis or anesthesia-related events. In these patients, NaviAid-assisted enteroscopy allowed for successful resection of hamartomatous polyps in the distal ileum, preventing surgery. Therefore given our experience with these cases previously presented in abstract form [6], NaviAid-assisted enteroscopy could be considered for resection of all small bowel polyps that cannot be resected via upper endoscopy or push enteroscopy.

## Conclusions

In conclusion, NaviAid-assisted enteroscopy provides another therapeutic option for removal of polyps without the use of specialized devices. NaviAid allows the endoscopist to make the decision to intervene on-the-spot when faced with unexpected findings such as deep hamartomatous polyps during routine colonoscopy. As a result, it prevents unnecessary and sometimes repetitive procedures for the patient. It is particularly useful in patients with small bowel lesions that require intervention.

Despite the advantages of NaviAid-assisted enteroscopy, it has yet to become well established in routine clinical practice. We suspect this is likely due to lack of awareness and limited number of studies establishing noninferiority of NaviAid to device-assisted enteroscopy. Based on our experience, and currently available data on NaviAid, we recommend additional consideration for the use of NaviAid-assisted enteroscopy in routine clinical practice for both antegrade and retrograde approaches to the small bowel. 
